# Reconstruction of local frequencies for recovering the unwrapped phase in optical interferometry

**DOI:** 10.1038/s41598-017-06801-z

**Published:** 2017-07-27

**Authors:** Julio C. Estrada, Jose L. Marroquin, Orlando M. Medina

**Affiliations:** 1 Centro de Investigacion en Matematicas (CIMAT), Computer Sciences, Guanajuato, 36023 Mexico; 2 Universidad Politecnica de Guanajuato (UPG), Robotics, Guanajuato, 38483 Mexico

## Abstract

In optics, when interferograms or digital holograms are recorded and their phase is recovered, it is common to obtain a wrapped phase with some errors, noise and artifacts such as singularities due to the non linearities of the demodulation process. This paper shows how to reconstruct the frequency field of the wrapped phase by using adaptive Gabor filters. Gabor filters are Gaussian quadrature filters tuned in at a certain frequency. We adapt these Gabor filters by tuning them locally and estimating the frequency using wrapped finite differences of the estimated phase. Doing this process iteratively, the frequency estimation is refined and smoothed. The unwrapped phase is easily recovered by integrating the recovered frequency field using, for example, a simple line raster integration. We don’t have problems with phase inconsistencies or residues while integrating the phase, because these are removed. The obtained unwrapped phase is clean, consistent and practically error-free. We show estimation errors with simulated data and the performance of the proposed method using real-world recorded wavefronts.

## Introduction

Today, in optical interferometry one can find dozens of papers showing how to recover the wrapped phase from a single interferogram with or without frequency carrier, or by using two or more phase-shifted interferograms, see refs 1–3 for a review. When the interferograms are free of noise, have high contrast and homogeneous illumination, these demodulation methods recover an ideal wrapped phase^4–6^. However under real laboratory conditions, for example when using speckle interferometry or shearography, we are far from these ideal conditions^7–12^. Instead, the wavefront recovered is a wrapped phase with imperfections like noise, miscalibration errors and artifacts that introduce phase inconsistencies or residues (singularities). These inconsistencies make the phase unwrapping process difficult, because the unwrapping is an integration of wrapped phase differences and this integration becomes path-dependent^13–16^. For the wrapped phase to be consistent, its wrapped differences must obey the following second order cross difference relation:

1 $${{\rm{\Delta }}}_{y}[{{\rm{\Delta }}}_{x}[{\varphi }_{w}]]={{\rm{\Delta }}}_{x}[{{\rm{\Delta }}}_{y}[{\varphi }_{w}]]$$

where Δ_*x*_ and Δ_*y*_ are the wrapped difference operator along *x* and *y* respectively. The wrapped difference operator, at a certain site *x*, is defined as the modulus 2*π* of the phase difference between the phase at *x* and its neighbor *x* − 1, assuming backward differences. When the wrapped differences obey Eq. (1), the phase unwrapping process is path-independent. The question is how can we estimate the wrapped differences in such a way that inconsistencies or residues are removed. What we are going to propose in this paper is based on the reconstruction of the noise-free frequency field, which is taken as the wrapped differences of the phase along *x* and *y*. Thus, given the wrapped phase with noise and miscalibration errors, we want to obtain the best possible estimation of the frequency field without noise and inconsistencies, in such a way that its integration becomes path-independent, so that one is able to unwrap the phase with simple line scanning integration.

There are two published methods that are related to the approach presented here. One is the work of ref. 16 in which the residual error between the observed wrapped phase and an estimated unwrapped and re-wrapped one, is iteratively approximated using a smoothed frequency field (where the smoothing is performed using a Total Variation (TV) scheme) which is then integrated to obtain the phase. This permits one to obtain a good approximation to the unwrapped phase, although the unwrapped phase is recovered with noise and inconsistencies. The other is the work of ref. 17 in which an estimation for the frequency field of an observed interferogram is obtained via a fixed bank of Gabor quadrature filters which filter the noise, and permit the estimation of the local frequency as the tuning frequency of the filter with maximal response. These two methods will be analyzed in more detail in the Discussion section.

In the method presented here, we also focus in the processing of the frequency field. The main difference in our work is that we propose the use of a linear filter whose tuning frequency is continuously adapted to the local frequency field, which allows for a better noise elimination, so that stable results may be obtained at very low signal to noise ratios. In particular, our proposal is to use a linear system whose impulse response is a Gabor filter tuned at the local frequency that is obtained from the wrapped phase. In the following sections we will show how the frequency field is estimated and used to tune the Gabor filters at each pixel and how the frequency reconstruction and smoothing is accomplished by using an iterative process involving the application of the adaptive Gabor filters and a smooth estimation of the frequency field using wrapped differences. We remark that just a few iterations of this process are needed to obtain a clean and consistent frequency field. Finally, the unwrapped phase is easily obtained after integration.

## Method

Let us start with the model of a typical wrapped phase obtained after demodulation:

2 $${\varphi }_{W}(x,y)=([\varphi (x,y)+c\,\sin \,\mathrm{[2}\varphi (x,y)]+\eta (x,y)]\,{\rm{mod}}\,2\pi )-\pi ,$$

where *ϕ*_*W*_(*x*, *y*) is the obtained wrapped phase, *ϕ*_*W*_(*x*, *y*) is the ground-truth phase or modulated phase, *c*sin[2*ϕ*(*x*, *y*)] is the miscalibration or tune-in error introduced by the demodulation algorithm used, and *η*(*x*, *y*) the induced noise. The noise is not necessarily normally distributed, for example in the case of speckle noise produced by coherent sources. The constant value *c* > 0 is directly proportional to the tune-in error of the algorithms used to demodulate the phase^18^. As the phase is affected by noise and wrapped modulus 2*π*, if Δ[*ϕ*(*x*, *y*) + *η*(*x*, *y*)] > |*π*| its wrapped differences do not obey the cross differences Equation (1), due to aliasing, and it translates to singularities and phase inconsistencies that difficult the phase unwrapping^13^. One must get rid of the noise and miscalibration error in order to obtain a perfect frequency reconstruction and phase unwrapping. The idea we use here is to apply an adaptive linear system to the following complex signal constructed from the wrapped phase

3 $$g(x,y)=\exp [i{\varphi }_{W}(x,y)],$$

where $i=\sqrt{-1}$. The frequency field *ω*(*x*, *y*) = [*u*(*x*, *y*), *v*(*x*, *y*)] for this complex signal can be obtained from the wrapped differences of *ϕ*_*W*_(*x*, *y*) as:

4 $$\begin{array}{rcl}u(x,y) & = & {{\rm{\Delta }}}_{x}[{\varphi }_{W}(x,y)]\\ v(x,y) & = & {{\rm{\Delta }}}_{y}[{\varphi }_{W}(x,y)],\end{array}$$

where the wrapped phase difference Δ_*x*_ and Δ_*y*_ can be given as

5 $${{\rm{\Delta }}}_{x}[\varphi (x,y)]=([\varphi (x,y)-\varphi (x-1,y)+\pi ]\quad {\rm{mod}}\,2\pi )-\pi $$

and

6 $${{\rm{\Delta }}}_{y}[\varphi (x,y)]=([\varphi (x,y)-\varphi (x,y-1)+\pi ]\quad {\rm{mod}}\,2\pi )-\pi .$$

The complex signal *g*(*x*, *y*) inherits the imperfections of the wrapped phase *ϕ*_*W*_(*x*, *y*) such as the noise and tune-in errors. To remove them effectively, it is not sufficient to use a linear shift-invariant system. We need an adaptive system that controls its tuning frequency for each site (*x*, *y*) and removes the phase noise letting pass only the local frequency of that site. To this end, we propose to use Gabor filters, which are defined as

7 $${h}_{\sigma ,{\omega }_{0}}(x,y)=\exp (-\frac{{x}^{2}+{y}^{2}}{2{\sigma }^{2}})\exp [-i{\omega }_{0}\cdot (x,y)],$$

where (*x*, *y*) is the spatial domain, *σ* controls the bandwidth and *ω*_0_ = (*u*_0_,*v*_0_) is the tuning frequency. The corresponding frequency response of the filter is:

8 $$H{(u,v)}_{\sigma ,{\omega }_{0}}=\exp (-\frac{{(u-{u}_{0})}^{2}+{(v-{v}_{0})}^{2}}{2{\sigma }_{\omega }^{2}}),$$

where *σ*_*ω*_ = 1/*σ*. Applying this filter in the frequency domain is equivalent to the convolution of the complex signal (3) with kernel (7), so that its output is expressed as

9 $$f(x,y)=\sum _{w,z}g(x-w,y-z){h}_{\sigma ,{\omega }_{0}}(w,z).$$

**Figure 1:**
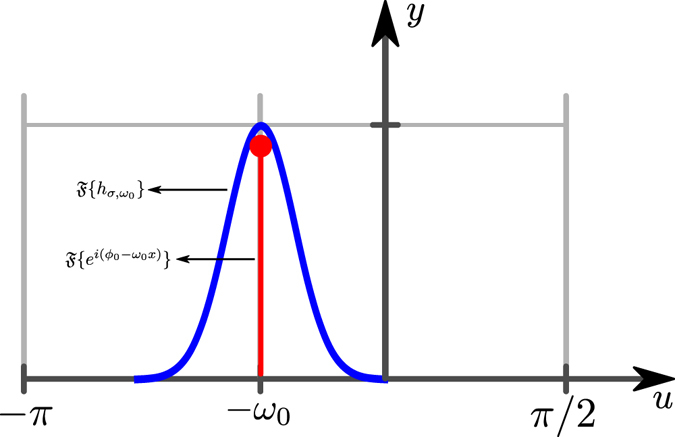
The red line represents an impulse at frequency *ω*_0_, which is the Fourier transform of a complex signal whose phase is a line with slope *ω*_0_. The blue line is the one-dimensional Fourier transform of the Gabor filter given by Eq. (7). The Gabor filter is tuned (or adapted) at the frequency *ω*_0_, passing only the information around this frequency *ω*_0_.

The standard application of this Gabor filtering, as depicted by this last equation, is to choose a tune-in frequency *ω*_0_ and apply the filter to all the image, i.e., the filter is shift-invariant. However, if we consider the first order local approximation around a site (*x*_0_, *y*_0_) of the signal phase, it is a plane with local frequencies (slopes) *u*_0_ and *v*_0_. Around this site (*x*_0_, *y*_0_), its Fourier transform is an impulse located at frequency *ω*_0_ = (*u*_0_, *v*_0_), as illustrated in one-dimension in Fig. 1. What we want is to adapt to this site (*x*_0_, *y*_0_) the Gabor filter according to its local frequency, in such a way that we are letting pass only the frequency information around *ω*_0_ = (*u*_0_, *v*_0_), as shown in Fig. 1. Then, for each pixel (*x*_0_, *y*_0_) we have an *ω*_0_ = (*u*_0_, *v*_0_) and the output is

10 $$f({x}_{0},{y}_{0})=\sum _{w,z}g({x}_{0}-w,{y}_{0}-z){h}_{\sigma ,{\omega }_{0}}(w,z);$$

where the tune-in frequency *ω*_0_ = (*u*_0_, *v*_0_) of the Gabor filter is not constant, but depends on the site (*x*_0_, *y*_0_), i.e., the filter is not shift-invariant. In this way, we are filtering out noise in an adaptive way.

### Reconstruction process

Given the wrapped phase *ϕ*_*W*_, which we assume is modeled as in (2), we take the first approximation of the frequency field as in Eq. (4), which give us the local frequency for each pixel (*x*, *y*) of the wrapped phase image. Then, we apply the adaptive Gabor filter using Eq. (10), so that for each site (*x*_0_, *y*_0_) the complex signal *g*(*x*_0_, *y*_0_) is convolved with its own Gabor filter tuned in at *ω*_0_ = [*u*(*x*_0_, *y*_0_), *v*(*x*_0_, *y*_0_)]. Then, we take the argument of the complex output *f*(*x*, *y*) to obtain a filtered phase as

11 $$\hat{\varphi }={\rm{\arg }}[f(x,y)].$$

An improved frequency field estimation is obtained as

12 $$\begin{array}{rcl}u(x,y) & = & {{\rm{\Delta }}}_{x}[\hat{\varphi }(x,y)]\\ v(x,y) & = & {{\rm{\Delta }}}_{y}[\hat{\varphi }(x,y)].\end{array}$$

As we are processing the wrapped phase corrupted by noise and miscalibration errors, phase differences increase the noise power and introduce singularities with frequency jumps of 2*π*. Therefore, it is necessary to filter the estimated frequency field so that these noise-induced inconsistencies are removed. This may be achieved by low-pass filtering the current local estimates given by Eq. (4), using a Gaussian kernel. In particular we compute the filtered frequency estimates as:

13 $$\begin{array}{rcl}\hat{u}(x,y) & = & \sum _{\forall (m,n)}u(m,n)\exp [-\frac{{(n-x)}^{2}+{(m-y)}^{2}}{2{s}^{2}}]\\ \hat{v}(x,y) & = & \sum _{\forall (m,n)}v(m,n)\exp [-\frac{{(n-x)}^{2}+{(m-y)}^{2}}{2{s}^{2}}].\end{array}$$

To set the *s* parameter, one may consider that if *F*_*M*_ is the maximum frequency magnitude that one wants to preserve in the spatial variation of the frequency fields, then the frequency response of this smoother should be such that at *F*_*M*_ one does not get too much attenuation. In particular, we set this attenuation so that the *s*_*ω*_ parameter of the Gaussian frequency response of the smoother is *s*_*ω*_ = *F*_*M*_/3, which in the space domain gives *s* = 3/*F*_*M*_. We have found experimentally that for a sample of images with a resolution of 200 × 200 pixels, the best value for *F*_*M*_ is about *π*/4.2. We also found that using the same value for the *σ* parameter for the adaptive Gabor filter gives good results; we note that the precise value for these parameters is not important for the performance of the method, as long as it is within the appropriate range. If the resolution of the images vary, however, one must make a correction, since the frequency content of the phase image scales linearly with the resolution (taken here as the width *N* of the image). Therefore, one gets the following formula for the parameter values, which is the one we use in all the experiments presented here:

14 $$s=\sigma =\frac{3\times 4.2\times N}{200\pi }.$$

The frequencies *û*(*x*, *y*) and $\hat{v}(x,y)$ represent a clean and refined estimation of the frequency field. Thus, we can use them again to improve the output *f*(*x*, *y*), obtaining a cleaner phase $\hat{\varphi }(x,y)$ and estimate again a smooth frequency field $\hat{u}(x,y)$ and $\hat{v}(x,y)$ from this using (13). After few iterations of this process, the obtained smooth frequency field $\hat{u}(x,y)$ and $\hat{v}(x,y)$ is integrated to obtain the unwrapped phase using a simple line scanning as follows:

- First set *ϕ*(0, 0) = 0. Then integrate line 0 using the estimated frequency $\hat{u}(x,y)$:
  15 $$\varphi (x,y)=\varphi (x-1,y)+\hat{u}(x,y)$$
  starting with *x* = 1.
- Then, integrate the remaining lines (starting with the line corresponding to *y* = 1) using the phase estimate at the previous line and the estimated frequency $\hat{v}(x,y)$:

16 $$\varphi (x,y)=\varphi (x,y-1)+\hat{v}(x,y)$$

**Figure 2:**
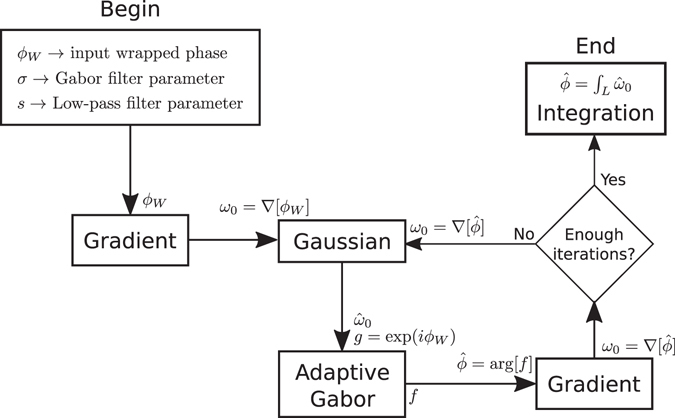
Block diagram of the method presented here. The gradient is taken by means of finite differences as in (4). Convergence takes about 6 iterations (see text).

The block diagram for the complete algorithm is shown in Fig. 2.

### Data Availability

In the results presented here, we use both simulated and real experimental data for the tests. For the simulated data the model for generating them is given in the description of each test. The real experimental data were obtained by O.M. Medina at the *Centro de Investigaciones en Optica* (CIO), Mexico, and may be available by requesting them directly to the authors.

## Results

For the simulated tests we present here, we take the Root Mean Square Error (RMSE) to measure the error as:

17 $${\rm{RMSE}}=\sqrt{\frac{1}{M\times N}\sum _{x,y}({[\hat{u}(x,y)-u(x,y)]}^{2}+{[\hat{v}(x,y)-v(x,y)]}^{2})},$$

where $\hat{u}(x,y)$, $u(x,y)$, $\hat{v}(x,y)$, and $v(x,y)$, are the frequency estimation and corresponding ground truth along *x* and *y*, respectively. *M* and *N* denote the number of rows and columns of the lattice (*x*, *y*) for the wrapped phase images.

**Figure 3:**
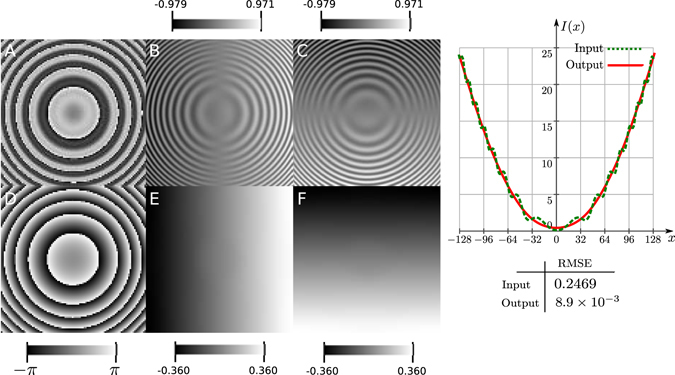
Wrapped phase with miscalibration errors. In (**A**) we see the wrapped phase, (**B** and **C**) show the *x* and *y* frequency fields. In (**D**). We show the recovered phase after frequency reconstruction and integration. The phase is shown wrapped for comparison proposes. In (**E** and **F**) we show the reconstructed frequencies. The graph at the right shows a plot of the central row of the unwrapped input and the unwrapped output of images (**A** and **D**) respectively. Color bars show the dynamic range of the images.

First we show how the method presented here removes the miscalibration errors. For example, in phase shifting interferometry, error sources of miscalibration are very common when the step of the phase shift is not given correctly or when the phase shifting algorithms are not tuned to the frequency step of the interferograms. We simulated a wrapped phase image using Eq. (2) without noise, taking $\varphi (x,y)=1.5\times {10}^{-3}((x-{\mathrm{128)}}^{2}+{(y-128)}^{2})$ in a lattice of *M* × *N* = 256 × 256 pixels and miscalibration error of *c* = 0.8. In theory, the frequency fields are two ramps, one in *x* and another in *y* with slope of 3 × 10^−3^ each one. Usin the Eq. (14), we set the filter parameters to *σ* = *s* = 5.5 for the Gabor and Gaussian filter, respectively, and performed 5 iterations. In Fig. 3(A) we show the simulated wrapped phase and Fig. 3(B,C) shows the frequency field taken as the wrapped differences of the simulated phase using Eq. (4). In Fig. 3(D) we show the obtained phase after frequency reconstruction and smoothing. The phase is recovered unwrapped but we show it wrapped for comparison purposes. The reconstructed frequency field is shown in Fig. 3(E,F). Compare the frequency field of B and C with the reconstructed frequency field of E and F in the figure. In B and C one can see the effect of the miscalibration error which is removed in E and F. The graph on the right, shows the central row of both unwrapped phases, the one with miscalibration errors and the one recovered after integrating the reconstructed frequency field. In the table shown below the graph, we can see that the miscalibration error is reduced by at least two orders of magnitude.

The second test shows how we can reconstruct the frequency field and obtain a clean unwrapped phase under different levels of noise, which we measure using the Signal to Noise Ratio (SNR). In this case, due to the noise, we split the iterations in two: first we perform 3 iterations with *σ* = 1.0 for the Gabor filter and *s* = 4.3 for smoothing the frequencies, then, we perform 3 more iterations setting *σ* = *s* = 4.3, according to Eq. (14), for doing a total of 6 iterations. We do it in this way because experimentally we found that for noisy phase maps one can obtain a better estimation for the local frequencies by relaxing the Gabor filtering with an small *σ* in order to get the detail of the local frequencies, then, set *σ* = *s* of the Gabor filter to filter out noise.

**Figure 4:**
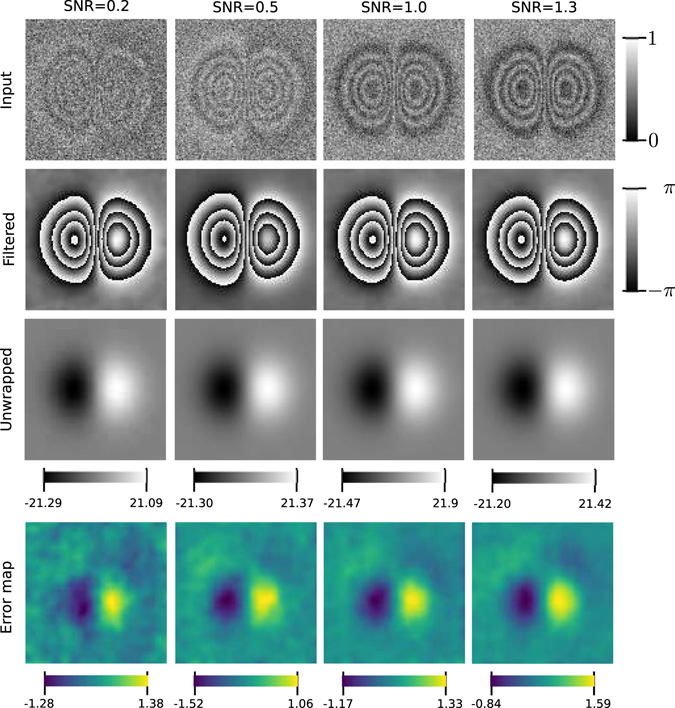
Test with four different levels of noise. The image columns show the test for SNR = 0.2, 0.5, 1.0 and 1.3 as depicted. The rows show the noisy fringe pattern, the obtained filtered phase, unwrapped phase and the error map. The color bars indicate the dynamic range of the images. The error map is the error (in radians) we obtain for each pixel by taking the difference of the obtained unwrapped phase and the ground true phase.

For this test, we simulated a wrapped phase as

18 $${\varphi }_{W}(x,y)={\rm{atan2}}(Is(x,y),Ic(x,y)),$$

where $Is(x,y)=\,\sin \,[\varphi (x,y)]+\eta (x,y)$ and $Ic(x,y)=\,\cos \,[\varphi (x,y)]+\eta (x,y)$. The scalar field *η*(*x*, *y*) is Gaussian noise with mean zero and variance *σ*_*n*_, the argument *ϕ*(*x*, *y*) is the ground true phase for which we take the same model used in ref. 16, which is:

19 $$\varphi (x,y)=\frac{2\pi }{\lambda }{\varphi }_{0}\exp [-({x}^{2}+{y}^{2}\mathrm{)/(2}{a}^{2})]$$

where *x*, *y* ∈ [−1, 1]^2^, and we take *ϕ*_0_ = 20 and *a* = 0.3, in a lattice of *M* × *N* *=* 201 × 201 pixels. The SNR of the signals *Ic* and *Is* is measured as 1/*σ*_*n*_ since the variance of the cosine and sine functions is one. As already said, the parameter *σ*_*n*_ corresponds to the variance of the noise, which is normally distributed. In Fig. 4 we show the result of this test for levels of SNR = 1.3, 1.0, 0.5 and 0.2. In row *Input* we show the noisy fringe pattern *I*_*c*_ that modulates the phase of Eq. (19), in row *Filtered* we show the wrapped phase obtained at end of the process iterations, in row *Unwrapped* we show the unwrapped phase we obtain after intergrating the reconstructed frequency field and row *Error map* shows the error obtained for each pixel as the difference between the obtained unwrapped phase and the ground true phase. Note that the error is localized mostly near the peaks of the phase, due to the reduction in dynamic range caused by the smoothing process; this reduction is in all cases less 10% of the total dynamic range of the unwrapped phase.

**Figure 5:**
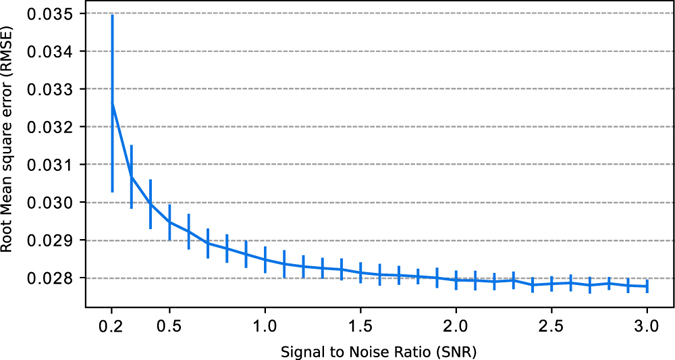
Graph of the Root Mean Square Error (RMSE) and standard deviation (vertical bars) for SNR levels from 0.3 to 3. The mean and standard deviation of the RMSE was taken from 100 different realizations for each level of SNR.

To see how accurate the reconstruction is, we used the same simulated phase of Fig. 4 and tested our method with 29 different levels of SNR from SNR = 0.2 to SNR = 3.0. For each level of noise, we ran 100 different realizations, reconstructed the phase and took the RMSE. The mean of the RMSE and standard deviation for each level of noise is shown in the error graph of Fig. 5. There we can see that the error is small and decreases proportionally with the level of noise. The variance of the RMSE let us see the stability of our method. For the case of SNR = 0.2, the variance is greater but consider that SNR = 0.2 is a very high leve of noise. These tests were made in a computer with 4xIntel^®^ Core i5-6300U CPU @ 2.4 GHz and 16 Gb of RAM. The mean processing time for each image was of 2.882 sec. The results shown here were obtained using double precision arithmetic.

### Special case: Phase Shifting Interferometry

In the examples we have presented, we assume that the wrapped phase map is the only data available, and that it follows the model of Eq. (2) and showed that our method obtains stable results for SNR >0.2, as shown in Fig. 5. However, in most cases we may have access to the interferograms from which the phase was obtained. We now show that in this case the noise tolerance of our method may increase. As an example, consider the case of Phase Shifting Interferometry (PSI). In this case, we have a sequence of interferograms which may be modeled as:

20 $${I}_{n}(x,y)=1+\,\cos \,[\varphi (x,y)+\alpha n]+noise(x,y),\quad n=0,1,2,\ldots ,N-1.$$

In particular, we consider the same example used in Fig. 6 of ref. 16, so that a comparison may be established between the performance of both methodologies. Therefore, as in ref. 16 we use *N* = 4, *α* = *π*/2, additive white Gaussian noise, and the same definition for the phase (see Eq. (19)), to obtain a four-step phase shifting sequence of interferograms. In this case, we can obtain a complex signal by combining them in the following way (derived from the standard 4-step phase recovery algorithm^8^):

21 $$g(x,y)={I}_{0}(x,y)-{I}_{2}(x,y)+i[{I}_{1}(x,y)-{I}_{3}(x,y)].$$

**Figure 6:**
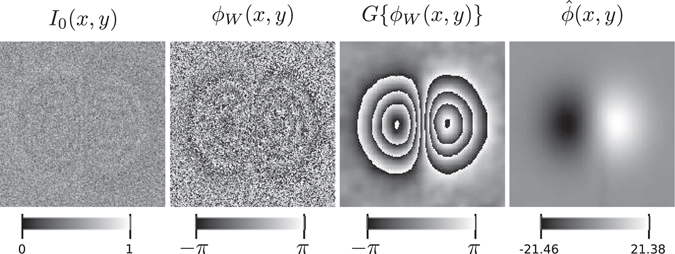
Result obtained by using the four interferograms of a four-step PSI sequence. The first image is the first fringe pattern of the 4-steps sequence; the second image is the wrapped phase obtained from the 4-step algorithm; the third image is the filtered wrapped phase we obtain, denoted here as *G*{*ϕ*_*W*_(*x*, *y*)}, and the fourth image is the corresponding unwrapped phase. The signal to noise ratio of interferograms is SNR = 0.08. The color bars indicate the dynamic range of the images.

Using this complex signal in our reconstruction method, instead of the complex signal of Eq. (3), the method is more robust and can deal with interferograms with SNR as low as 0.08, while in the case of the experiment reported in ref. 16, their method becomes unstable for SNR = 0.3. In Fig. 6, we show the first interferogram of the sequence described above with SNR = 0.08, which is lower than the SNR used in Fig. 4. Using the complex signal (21) and following our method, with the same iterations and parameters as described for the test of Fig. 4, we obtained a cleaner phase (comparing with the input). This is appreciated in the last two images of Fig. 6.

### Comparison with TV filtering approach

**Figure 7:**
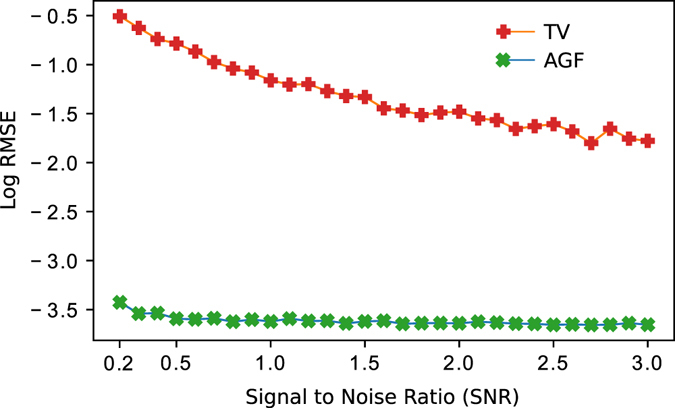
The graph shows the error obtained using the Adaptive Gabor Filters (AGF) approach, shown here, and the TV filtering approach shown in ref. 16. For clarity, the error is in logarithmic scale.

To perform a fair comparison with the work presented in ref. 16, we programmed that method as described in that work so that we could reproduce their results, and then compared the RMSE for as defined in Eq. (17) with our approach. We ran this test for 29 different levels of noise from *SNR* = 0.2, to 3.0 with steps of 0.1 with the same test image shown in Fig. 4. For each noised simulated wrapped phase, we applied the method described in ref. 16. and the one described here. In Fig. 7 we show the RMSE in logarithmic scale of both methods for each level of noise. We used the logarithmic scale for clarity. As one can see, with the approach presented one obtains a RMSE at least two orders of magnitude smaller than the one obtained with the approach presented in ref. 16 (see Discussion section).

### Tests with real data

In the last test, we used three different wrapped phases obtained from real optical experiments using speckle interferometry.

**Figure 8:**
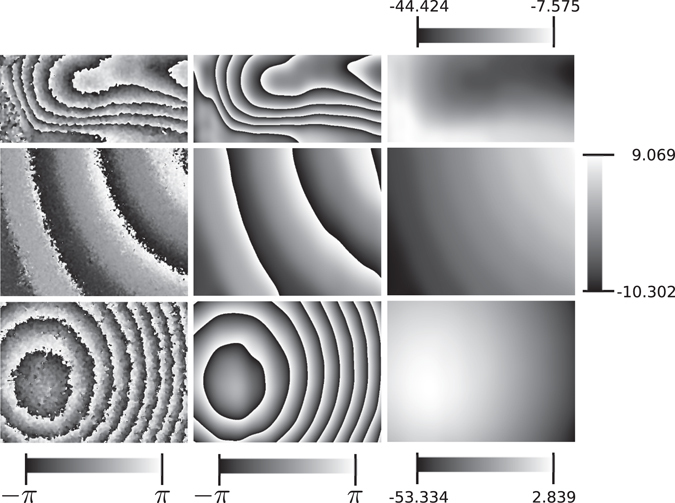
Test using real laboratory experiments. The first column shows the input, the second column shows the filtered wrapped phase and last column shows the corresponding unwrapped phase. All experiments are from speckle interferometry.

In Fig. 8 we show the results. The inputs for these tests are affected by speckle noise and miscalibration errors. The miscalibration error is evident in the test shown in the middle row if you compare how it is seen in Fig. 3. In the column *Reconstruction* we can see the recovered wrapped phase obtained by integrating the reconstructed frequency field. In the column *Unwrapped* we show the unwrapped phase obtained after integrating the frequency field. As one can see, the final unwrapped phase is obtained clean without detuning errors. The sizes of each wrapped phase image given as input are 246 × 527, 480 × 610 and 482 × 641, respectively. As with the test of Fig. 4, we made 6 iterations in total of our method. The first 3 iterations are with parameters *σ* = 1.0 and *s* = 11.3 and the next tree iterations are with parameters *σ* = *s* = 11.3. As before, the parameters *s* and *σ* are set according to Eq. (14), using N = 527, which is the width of the first wrapped phase of Fig. 8. Note that as we said, it is not necessary to use the precise values given by Eq. 14; the intermediate value we use gives good results in all these cases.

## Discussion

We have presented a simple and computationally efficient method for the accurate reconstruction of the frequency field from the noisy wrapped phase which is obtained from optical experiments. From this denoised frequency field the clean unwrapped phase may be easily obtained by path-independent integration.

**Figure 9:**
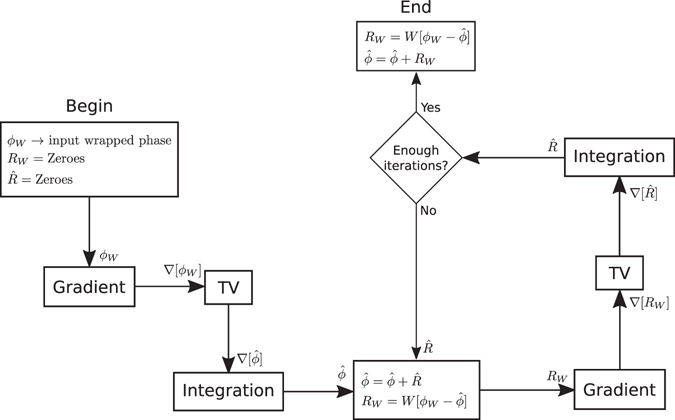
Block diagram of the method presented in ref. 16. As with our method, the gradient is taken by means of finite differences. As recommended in ref. 16. we use 500 iterations in the experiment reported here.

The basis of the method is the use of an adaptive linear quadrature filter which permits very good noise elimination. The method makes the implicit assumption that the true unwrapped phase is smooth. If this holds, one can obtain better denoising performance than that of even non-linear wide passband methods. This is important because, as we have seen here, the problem of phase unwrapping may be complicated due to the presence of noise which induces inconsistencies in the corresponding frequency field. In^16^, the approach is by approximating in an iterative way the residual error between the observed wrapped phase and an estimated one, which is obtained by integrating a TV-smoothed frequency field. To summarize the method we developed a block diagram for each iteration which is presented in Fig. 9. One problem with this method is that when one adds to the current unwrapped estimator ${\hat{\varphi }}_{W}$ the final estimated wrapped residual error *R*_*w*_ between the observed wrapped phase and the re-wrapped ${\hat{\varphi }}_{W}$, one effectively obtains an unwrapped phase that is appropriately unwrapped, but with the original (wrapped) noise added back to it. As a result, the performance of the method decreases sharply as the SNR diminishes. In contrast, in our approach all the noise is progressively eliminated from the estimated unwrapped phase by means of the adaptive Gabor filter, which explains the big difference in RMS error between the two approaches, which is observed in Fig. 7. It should be noted however, that for high SNR, the method in^16^ will give very good results because adding the final residual *R*_*w*_ permits the full preservation of the phase dynamic range, while in our approach it may be slightly compressed due to the smoothing process. For the same reasons, the method in ref. 16 may be more adequate for the unwrapping of discontinuous phase maps, since in our approach the discontinuities may be smoothed out to some degree.

Also related to the approach presented here is the work of ref. 17. There, a local frequency map is estimated by passing an observed interferogram through a fixed bank of Gabor filters and then estimating the local frequency as the tuning frequency of the filter with maximal response. Some problems with this approach are its sensitivity to noise and the fact that since the estimated frequency is restricted to the discrete set of tuning frequencies of the filter bank, one always gets a quantization error which is inversely proportional to the number of filters in the bank, and hence, to the computational complexity. In contrast, in our approach we start with the observed wrapped phase map and estimate the local frequency as the phase gradient; this estimate is progressively refined through the iterations of the method, and used to tune local Gabor filters that eliminate noise, until one finally gets a high-precision, smoothly-varying frequency map, which is then used to find a clean, inconsistency-free unwrapped phase map.

Our method may be very useful in optical applications, such as interferometry, shearography and digital holography, as well as in other areas, such as fringe projection for 3-D reconstruction of smooth objects. In many of these instances, the reconstruction problem is difficult due to the presence of speckle noise and miscalibration errors; as we have shown, our method presents an excellent performance in these cases. However, if there are many true phase discontinuities present, as is the case, for example, in Synthetic Aperture Radar (SAR) images of urban landscapes, other methods, perhaps based on the TV functional, or dedicated algorithms^19, 20^ may be more adequate.
